# Origin of congenital coronary arterio-ventricular fistulae from anomalous epicardial and myocardial development

**DOI:** 10.1038/s12276-022-00913-x

**Published:** 2023-01-18

**Authors:** P. Palmquist-Gomes, A. Ruiz-Villalba, J. A. Guadix, J. P. Romero, B. Bessiéres, D. MacGrogan, L. Conejo, A. Ortiz, B. Picazo, L. Houyel, D. Gómez-Cabrero, S. M. Meilhac, J. L. de la Pompa, J. M. Pérez-Pomares

**Affiliations:** 1 Department of Animal Biology, Faculty of Sciences, University of Málaga, 29071 Málaga, Spain; 2 IBIMA-Plataforma BIONAND (Junta de Andalucía, Universidad de Málaga), 29590 Campanillas (Málaga), Málaga, Spain; 3 Université de Paris, Imagine-Institut Pasteur, Unit of Heart Morphogenesis, INSERM UMR1163, 75015 Paris, France; 4 Advanced Genomics Laboratory, Program of Hemato-Oncology, CIMA, University of Navarra, Pamplona, 31008 Spain; 5 Université de Paris, M3C-Necker Enfants malades, AP-HP, 75015 Paris, France; 6 Intercellular Signalling in Cardiovascular Development and Disease Laboratory, National Centre of Cardiovascular Research-Instituto de Salud Carlos III, 28029 Madrid, Spain; 7 CIBER de Enfermedades Cardiovasculares, 28029 Madrid, Spain; 8 Hospital Materno-Infantil de Málaga, Instituto Malagueño de Biomedicina (IBIMA), 29080 Málaga, Spain; 9 Traslational Bioinformatics Unit, Navarrabiomed, Complejo Hospitalario de Navarra (CHN), Universidad Pública de Navarra (UPNA), IdiSNA, 31008 Pamplona, Spain; 10 Biological and Environmental Sciences and Engineering Division, King Abdullah University of Science and Technology, 23955 Thuwal, Saudi Arabia

**Keywords:** Embryology, Congenital heart defects

## Abstract

Coronary Artery Fistulae (CAFs) are cardiac congenital anomalies consisting of an abnormal communication of a coronary artery with either a cardiac chamber or another cardiac vessel. In humans, these congenital anomalies can lead to complications such as myocardial hypertrophy, endocarditis, heart dilatation, and failure. Unfortunately, despite their clinical relevance, the aetiology of CAFs remains unknown. In this work, we have used two different species (mouse and avian embryos) to experimentally model CAFs morphogenesis. Both conditional *Itga4* (alpha 4 integrin) epicardial deletion in mice and cryocauterisation of chick embryonic hearts disrupted epicardial development and ventricular wall growth, two essential events in coronary embryogenesis. Our results suggest that myocardial discontinuities in the embryonic ventricular wall promote the early contact of the endocardium with epicardial-derived coronary progenitors at the cardiac surface, leading to ventricular endocardial extrusion, precocious differentiation of coronary smooth muscle cells, and the formation of pouch-like aberrant coronary-like structures in direct connection with the ventricular lumen. The structure of these CAF-like anomalies was compared with histopathological data from a human CAF. Our results provide relevant information for the early diagnosis of these congenital anomalies and the molecular mechanisms that regulate their embryogenesis.

## Cardiology: How heart development goes astray

Insights from animal models highlight specific disturbances in early heart development as a likely point of origin for the formation of coronary artery fistulas (CAF), abnormal linkages between one of the coronary arteries and other regions of the heart. Children born with such defects are at heightened risk of serious heart problems. Using chick and mouse embryos, José Pérez-Pomares at the University of Málaga in Spain revealed a mechanism by which CAFs may arise in humans. Early in development, the heart muscle that forms the ventricle chambers undergoes a process of growth and compaction that transforms ventricular walls into thick and dense tissue layers. The researchers demonstrated that genetic or physical disruptions that interfere with this process give rise to CAF-like features, highlighting a promising strategy for modelling this congenital defect.

## Introduction

Congenital heart disease (CHD) affects around 1% of new-borns^1^, and may have a direct impact on cardiovascular performance during paediatric and adult life^2^. Despite the remarkable social impact of CHD, not much it is known about the embryological defects underlying the origin of many of these conditions. This lack of information on the cellular and molecular mechanisms that cause CHD limits our chances of discovering intrinsic and extrinsic factors that potentially disrupt normal embryonic heart development, and novel early diagnostic markers for these ailments^3^. We are thus still far from having enough information to design and implement an effective CHD prevention strategy. In this work, we provide experimental, mechanistic evidence on the origin of a distinct form of CHD that affects coronary blood vessels and other cardiac structures.

Coronary artery (CA) anomalies are a well-characterized subset of CHD^4^. Some CA anomalies can severely disrupt cardiac function and even result in sudden cardiac death. It has been reported that 0.6–1.5% of patients undergoing invasive cardiovascular imaging have coronary anomalies, including coronary artery fistulae (CAFs)^4–6^. CAFs are abnormal communications between a coronary artery and other cardiovascular cavity that often are asymptomatic, but can also cause long-term variations in the systemic hemodynamics, normally due to the arrest of part of the blood circulation that may result in ischaemia. The two main sub-types of CAFs described in the literature involve the connection of a coronary artery to either the lumen of a cardiac chamber (coronary-cameral fistula) or a large cardiac vessel (coronary arterio-venous fistula). The most common CAF type reported in the literature is the arterio-ventricular one (41%), followed by coronary artery connections to the right atrium (26%), the pulmonary trunk (17%), the coronary sinus (7%), the left atrium (5%), and the left ventricle (less than 3%)^7^. In particular, arterio-ventricular fistulae involving large coronary vessels often manifest as a coronary wall convexity (dilation) forming a conspicuous cameral, pouch-like structure at the cardiac surface. Although studies differ in their conclusions on CAF incidence in the right versus the left ventricle^7,8^, solitary ventricular CAFs were found to be more frequent than multiple microfistulas^9^.

It is well established that the vast majority of CAFs identified at birth have a congenital origin^10^. Importantly, these cardiac congenital defects should not be mistaken for isolated connections existing between blood vessels and /or cardiac cavities that result from surgical interventions, traumatic events, or infections (e.g., endocarditis), which are often referred-to as ‘fistulae’ and even ‘pseudoaneurisms’^11–13^. Indeed, congenital CAFs are true anomalies that derive from abnormal embryogenesis and, as such, they frequently associate to other forms of CHD, including Tetralogy of Fallot, atrial and ventricular septal defects (VSDs), persistent ductus arteriosus, pulmonary atresia and left ventricular non-compaction, among others^8^. Some authors have attempted to relate CAFs with gross genetic abnormalities such as chromosome deletions, e.g., 22q11.2^14^ and conditions involving chromosome number variation like Turner, Klinefelter, or Down syndromes^15^, but so far the pathogenesis of CAFs remains largely unknown.

Approaching the mechanistic origin of CAFs requires a detailed understanding of embryonic coronary vascular and ventricular wall formation and maturation. Recent studies have demonstrated that coronary arteries form from a primary endothelial capillary network, including cells from the endocardium (sinus venosus and ventricle), and the epicardial progenitors at the *septum transversum*/proepicardium (ST/PE)^16–18^. This finding implies that, in order to complete the coronary vascular circuitry, cells from the inner ventricular endocardium (preferentially fated to form coronary arteries and capillaries) and the outer epicardium (comprising endothelial cells that will mostly incorporate to coronary veins), merge by crossing the cardiac chamber myocardium that separates them. Moreover, the epicardium, whose contribution to the forming coronary vasculature (intimal endothelium, medial smooth muscle cells and adventitial fibroblasts) is well-known^18–21^, also acts as an instructive signalling centre that promotes the growth and the maturation of the adjacent myocardium^22^. Therefore, it is very likely that both anomalies in the cells that build the coronary vascular tree and the disruption of myocardium growth and maturation are involved in the origin of CA anomalies. In accordance with this hypothesis, animal models for defective epicardial development display a thin compact ventricular myocardium^23^ and coronary defects^24,25^, some of which are reminiscent of CAFs^26^.

In this work, we hypothesize that the disruption of embryonic ventricular compact myocardial growth leads to the formation of CAF in both mammals and avians. Compact myocardium growth arrest in mouse embryos is achieved indirectly, i.e., by disrupting epicardial formation and the subsequent depletion of the epicardial-derived pro-mitotic factors that regulate cardiomyocyte proliferation. To interfere with murine epicardial development, we have conditionally deleted the *Itga4* (*α4-integrin*) gene in epicardial progenitors. *Itga4* encodes for the α4 integrin subunit, whose cardiac expression is restricted to the epicardium. Integrin dimers including α4 integrin subunit promote epicardial adhesion to the myocardium by interacting with the myocardial-expressed Vcam-1 ligand. Since mice with systemic *Itga4* loss-of-function show an extreme phenotype and die around embryonic day (E) 9.5–10^27^, we have used the *Cre/LoxP* system to circumvent this early embryonic lethality while preserving the anomalous epicardial formation and the myocardial defects that are secondary to epicardial developmental anomalies. In avian embryos, local loss of myocardial integrity is directly achieved by cryocauterisation of the ventricular wall without the involvement of primary epicardial defects^26^. To uncover the cellular and molecular mechanisms underlying CAF pathogenesis, transcriptomic analysis of mutant mice and careful immunohistochemical characterization of *Itga4* mutants and cryocauterised avian embryonic hearts have been carried out. Finally, results from these experimental studies were compared with histopathological data from a human paediatric CAF to confirm our experimental findings.

## Methods

All animals used in our research program were handled in compliance with the European and Spanish *guidelines for animal care and* welfare.

### Mice

To circumvent the early lethality of *Itga4* systemic ablation^27,28^, this gene was conditionally deleted in the ST/PE by using a *G2-Gata4-Cre* mouse transgenic line^18^. *Itga4*^flox^ and *G2-Gata4*-Cre mice have been previously described^29,30^. To track epicardial derivatives, *G2-Gata4*-Cre mice were also crossed with *Rosa26R*-YFP mice^18^. Heterozygous *G2-Gata4*^Cre/+^ were bred with Itga4^flox/+^ mice to generate *G2-Gata4*^Cre/+^*;Itga4*^flox/flox^ mutants. (i.e., substitute animals for mutants).

#### In situ hybridization (ISH)

E9.5 embryos were fixed, embedded in paraffin, and sectioned in sterile conditions. In situ hybridization was performed following standard protocols (see supplementary information). The probe was generated with the forward 5′-ATGGTAACCGTAGCTGTACCT-3′ primer and the reverse 5′-AGTCATCCTTGTTCCCACTTG-3’ primer.

#### Trichrome staining of paraffin-embedded samples

For routine histological analyses, embryos or embryonic hearts were fixed, embedded in paraffin, and sectioned. Tissue sections were dewaxed in xylene, hydrated in ethanol series, and rinsed in distilled water. Samples were then submitted to Mallory’s trichrome staining (see supplementary information).

### Immunohistochemistry in mouse embryos

For immunohistological analyses, embryos were fixed in a 4:1 methanol:DMSO solution overnight at −20 °C, dehydrated in an ethanolic series, embedded in paraffin, and sectioned in a microtome. Immunofluorescence of the tissue was performed following standard methods (see Supplementary Information). Primary and secondary antibodies used in this study are listed in Supplementary Tables 1 and 2, respectively.

### BrdU tissue incorporation, immunohistochemistry, and quantification

A working volume of 420–450 µl from an aqueous BrdU (Sigma, B9285) stock solution (10 mg/mL) was injected intraperitoneally in pregnant females 30 min before embryo extraction. Embryos were fixed in a 4:1 methanol:DMSO solution overnight, embedded in paraffin, and sectioned with a microtome. Sections were dewaxed, rehydrated, treated with 2 N HCl (30′ at room temperature), and washed again in 100 mM sodium tetraborate (5′). Following steps and image acquisition were performed according to standard protocols (see supplementary information). Proliferating cardiomyocytes were estimated with the IMARIS® software (see supplementary information). A t-test and a post hoc Lubischew coefficient analyses were used to assess statistical significance.

### RNA sequencing (RNA-Seq)

RNA was isolated from heart ventricles of *G2-Gata4*^+/+^*;Itga4*^flox/+^ control and *G2-Gata4*^Cre/+^*;Itga4*^flox/flox^ mutant E11.5 embryos using the Arcturus Picopure RNA isolation kit (Applied Biosystems), including a step for removing genomic DNA. For each genotype, RNA coming from 3 ventricles was pooled (*n* = 4–5). Quality control was performed on an Agilent 2100 bioanalyser using an RNA 6000 Pico LabChip kit (Agilent Technologies). The RNA integrity number (RIN) was 10 for all the samples. cDNA was prepared using the standard Illumina TrueSeq RNASeq library preparation kit. Libraries were sequenced in a GAIIx Illumina sequencer using a 75 bp single end elongation protocol. Sequencing read quality was assessed with FastQC (S. Andrews, Babraham Institute). Contaminating Illumina adapters were trimmed with Cutadapt 1.7.1 which also discarded reads that were shorter than 30 bp. The resulting reads were mapped against the mouse transcriptome (GRCm38, release 76) and quantified using RSEM v1.2.20.

Prior to statistical analysis, genes with sufficiently large counts were identified and retained with the function filterByExpr, implemented in the R package edgeR. Data normalization and statistical testing was performed with DESeq2. Enrichment analyses for signalling pathways and gene ontology categories were performed with the R package clusterProfiler. KEGG was selected as the database for pathways and we focused on biological process ontology.

#### qPCR validation and data analysis

RNA isolation and qPCR analyses were performed following routine protocols (see Supplementary Information). The melting curve analysis (ViiA TM 7 Real-Time PCR system, Applied Biosystems) and size fractionation by agarose gel electrophoresis were used to confirm amplification of the expected products. Primers used are listed in Supplementary Table 3. Target quantity (N0) was obtained from the extracted raw data using LinRegPCR program. *Pgk1* and *Ppia* were selected as reference genes as previously described^31^. Primer sequences (Supplementary Table 3) were designed using primer3, BLAST (NIH) and oligo analyzer (IDT) software. Graphs and statistical analysis were performed by a non-parametric one-way analysis of variance with a Kruskal-Wallis post-hoc test in GraphPad Prim version 6.0 (GraphPad Software) (*P* < 0.05).

### Protein–protein interaction (PPI) models

A PPI sub-network was identified by selecting genes derived from the RNA-seq data analysis, then identifying the predicted proteins associated with these genes and selecting genes and interactions within the first two levels of interaction (A-B-C), considering up to 10 contacts per level (see Supplementary information for further details). As a result, two different sub-networks were identified, one from *VCAM1* and the second from *ITGA4*, representing two different cellular types, cardiomyocytes, and epicardial cells, respectively. Interactions between proteins were downloaded from StringDB and visualized in Cytoscape. Nodes were selected based on first and second PPI associations with *VCAM1* and *ITGA4* using a maximum of 10 connections derived from “experiments” or “databases”.

### Cryoinjury of the avian embryonic heart

The second experimental model used in this work was the cryocauterisation of the avian embryonic heart. Chick and quail embryos were staged according to the Hamburger and Hamilton stages of chick development^32^ and the classification for quail developmental stages^33^, respectively. Eggs were kept in a rocking incubator at 38 °C for 55–60 h (early HH17). A N_2_-cooled copper probe was used to burn the myocardial wall as previously described^34^, In every single case, the embryos were inspected to ensure that epicardial cell progenitors (proepicardial cells) had not yet attached to the myocardial surface. Routine histological analyses were performed as previously explained for mouse embryos (Mallory’s trichrome staining).

### 3D reconstructions of chick-damaged hearts

To acquire 3D images, chick hearts were washed in PBS and fixed in paraformaldehyde for several weeks at 4 °C. Then, hearts were washed in methanol series, embedded in JB4 resin, sectioned and analyzed by a high resolution episcopic microscope (HREM). Two different stages (4 days post-injury “dpi” and 16 dpi) were analyzed. The 3D shape of the tissue was reconstructed using the ImageJ^®^ software.

### Immunohistochemistry in avian embryos

Chick and quail embryos were fixed in a 4:1 methanol:DMSO solution overnight, dehydrated in an ethanolic series, embedded in paraffin and sectioned in a microtome. Immunostaining of avian tissues was performed following the same protocol described in the mouse (see supplementary information). For QCPN immunostaining, epitopes were unmasked using TEG buffer (0.12% Trizma base and 0.02% EGTA diluted in distiller water; pH 9) in a pressure cooker for 10 min, and the signal was amplified with TSA biotin system kit (PerkinElmer, NEL700A001KT).

### Proepicardium-endocardium co-cultures

Sharp tungsten needles, small iridectomy forceps, and scissors were used to isolate quail tubular hearts (HH 16–17) under a dissecting scope. Isolated ventricles and proepicardia were cultured in different conditions (see Supplementary Data). All samples were fixed overnight in Methanol-DMSO (4:1) solution. Fixed tissue constructs were dehydrated in an ethanol series, washed in butanol, embedded in paraffin, and sectioned in a microtome. Some aggregates were not embedded in paraffin and processed as whole mount samples.

For immunohistochemistry, sections were stained following the same protocol than previously described for mouse samples (see Supplementary Information).

### Necropsy and histological inspection of a human paediatric CAF

The autopsy of a six weeks-old patient with a diagnosis of complex congenital heart disease, who had previously undergone a palliative procedure (Blalock–Taussig shunt) and had died of sudden cardiac arrest, was performed at the Necker Hospital (Paris, France). Adequate informed consent has been obtained for the study of paediatric tissue samples. Post-mortem analysis revealed massive cardiac tamponade as a probable cause of death. Formalin-fixed samples were embedded in paraffin, sectioned with a microtome, and mounted on slides. Tissue sections were dewaxed with xylene and hydrated in an ethanolic series. Routine histological analyses were performed by Mallory’s trichrome staining as detailed above. Immunochemical analyses of the human tissue were performed as described in the supplementary information.

## Results

### G2-Gata4^Cre^;Itga4^flox^ mutant mice display epicardial and myocardial developmental defects

**Fig. 1:**
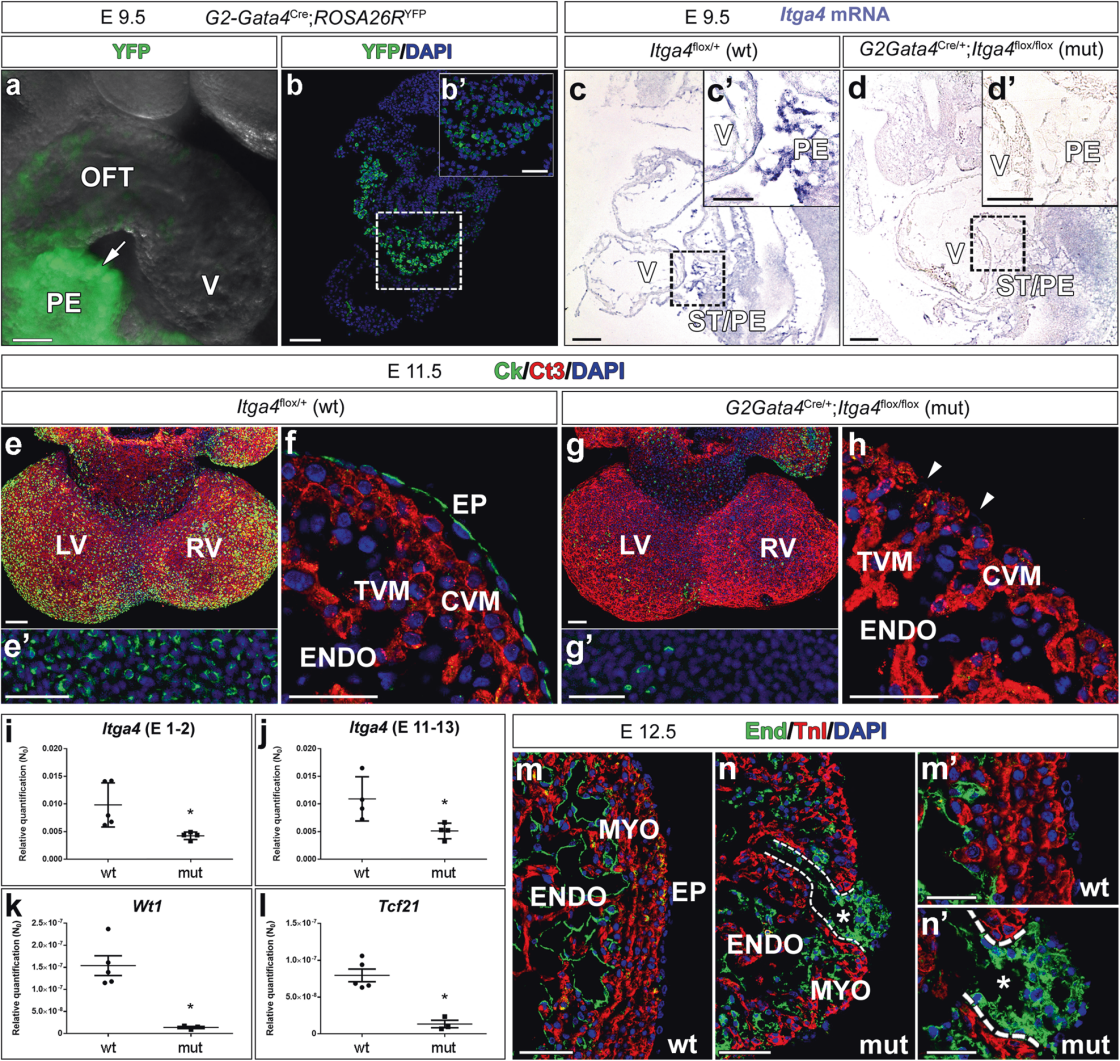
*Itga4* (α-4 integrin) disrupts epicardial and myocardial embryonic development and results in endocardial extrusions. **a**, **b** YFP reporter expression in *G2-Gata4-Cre;Rosa26R*-YFP mice highlights epicardial progenitor cells at the *septum transversum*/proepicardium (green fluorescence, arrow in **a**, boxed area in **b**−**b’**). **c, d** *G2-Gata4-Cre-mediated* *Itga4* deletion in the ST/PE severely reduces *Itga4* mRNA expression in mutant (*G2-Gata4*^Cre/+^;*Itga4*^flox/flox^) proepicardium (compare the boxed areas in **c**, **c’** and **d**, **d’**). E11.5 mutant mice display impaired epicardial (Ck^+^, green fluorescence) development (compare **e**, **f** with **g**, **h**). Ct3 counterstains the myocardium. Ventricular myocardial discontinuities are frequently found in mutant embryos (h, arrowheads). **i**–**l** RT-qPCR shows a significant (*p* < 0.05) loss of *Itga4* gene expression in E11.5 mutant ventricles (mut; *n* = 4) when compared to control ones (*n* = 5 in **I**; *n* = 4 in **J**), (**i**, **j**). The expression of the epicardial marker genes *Wt1* (k) and *Tcf21* (**l**) is also sharply reduced in mutant ventricles (*n* = 4) as compared to control ones (*n* = 5) (*p* < 0.05). Each replicate represents a pool of three ventricles. Statistical significance was obtained by a non-parametric one-way analysis of variance with a Kruskal–Wallis post hoc test. **m**, **n** At E12.5, the mutant ventricular endocardium (endomucin^+^) locally reaches the pericardial cavity and forms vesicular structures (asterisks indicate the inner lumen, compare **m**, **m’** with **n**, **n’**). The dashed line in **n**, **n’** marks the endocardial extrusion path through a myocardial discontinuity. The ventricular lumen is in communication with the lumen of the endocardial vesicle. Abbreviations: Ck cytokeratin; Ct3 cardiac troponin T; CVM compact ventricular myocardium; DAPI diamidino-2-phenylindole; End endomucin; ENDO endocardium; EP epicardium; LV left ventricle; PE proepicardium; PRC pericardial cavity; RV right ventricle; ST/PE *Septum transversum*/proepicardium; TnI troponin I; TVM trabeculated ventricular myocardium; V ventricle; YFP yellow fluorescent protein. Scale bars: **a**–**e**, **g**: 100 µm; **b**’–**g**’, **h**, **m**, **n**: 50 µm; **m**’, **n**’: 25 µm.

To evaluate the role of the epicardium in the origin of CAF, the *G2-Gata4-Cre* epicardial driver mouse line, displaying a strong Cre expression in ED 9.5 epicardial progenitor (proepicardial) cells (Fig. 1a, b), was crossed with mice carrying floxed *Itga4* alleles (*Itga4*). Crossing these two transgenic lines resulted in the conditional deletion of the *Itga4* gene in epicardial progenitors (*G2-Gata4*^*Cre/+*^;*Itga4*^*flox/flox*^, from here onwards, *G2-Itga4* mutants), as shown by the reduced *Itga4* mRNA levels found in *G2-Itga4* mutant proepicardia (Fig. 1c, d). E10.5 *G2-Itga4* mutant embryos did not show any evident myocardial wall anomaly, but epicardial formation, highlighted by the presence of cytokeratin-positive cells over the heart surface, was delayed in these animals as compared to stage-matched control embryos (Supplementary Fig. 1a, b). Epicardial developmental arrest continued to be evident in E11.5 (Fig. 1e–h) *G2-Itga4* embryos. At this stage, the compact ventricular myocardium remained abnormally thin in mutant embryos, which displayed discontinuities in their ventricular walls (Fig. 1g, h; Supplementary Fig. 2a). In accordance with the low numbers of epicardial cells found in *G2-Itga4* mutant embryos, the expression of *Itga4* and epicardial-related genes like the *Wilms’ tumour suppressor 1* (*Wt1*) and the transcription factor *Tcf21* was sharply reduced in mutant ventricles as compared with control ones (Fig.1i–l). This E11.5 epicardial growth arrest was evidenced by the absence of Wt1 protein on the heart surface (Supplementary Fig. 1c, d). At E12.5, *G2-Itga4* mutant embryos often presented evident pericardial haemorrhage, as shown by gross anatomical and histological examinations (Supplementary Fig. 1e–g). The ventricular myocardial discontinuities found in the ventricles allowed for the local extrusion of the endocardium (endomucin staining) towards the pericardial cavity forming characteristic pouch-like structures (Fig. 1m, n). Immunostaining confirmed the efficient deletion of *Itga4* in the epicardium of mutant embryos (Supplementary Fig. 1h, i’). At E13.5, all mutant embryos retrieved were dead.

### Epicardial Itga4 deletion alters the transcriptome of developing cardiac ventricles

**Fig. 2:**
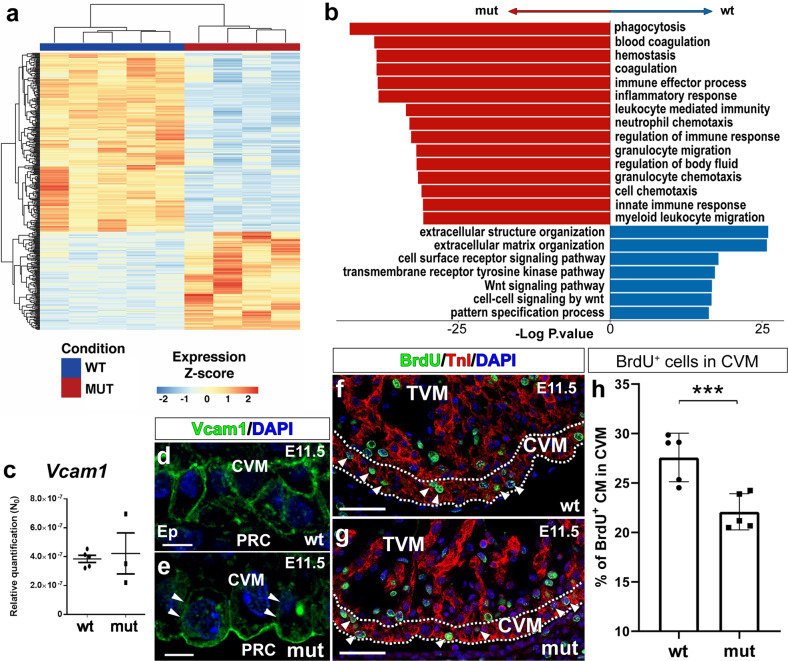
*Itga4* epicardial deletion modifies the transcriptomic and structural profile of embryonic ventricles. **a** Comparative RNA-seq analysis shows that 376 genes are differentially expressed (DEG) between mutant (*G2-Gata4*^Cre/+^;*Itga4*^flox/flox^) (*n* = 4) and control (*G2-Gata4*^+/+^;*Itga4*^flox/+^) (*n* = 5) E11.5 ventricles (*p*.value < 1e^−3^); 134 genes are increased and 242 are decreased. Each replicate represents a pool of three ventricles. **b** Gene Ontology (GO) enrichment analysis (an Over Representation Analysis with a Hypergeometric distribution with a Benjamini–Hochberg adjustment for multiple comparison) reveals important differences between the two genotypes. Pathway enrichment is expressed as the –log [P] adjusted for multiple comparison. The direction of the bars indicates which category was over/underrepresented. All categories in red are overrepresented in mutant animal, whereas all categories in blue are overrepresented in control animals. RT-qPCR analysis of E11.5 embryonic ventricles (*n* = 5 in control and *n* = 3 in mutant; each replicate represents a pool of three ventricles) does not reveal significant variations in *Vcam1* gene expression (**c**), but Vcam1 protein mislocalisation is evident in ventricular cardiomyocytes (compare **d** with **e**; arrowheads mark the reduced Vcam1 distribution in the lateral plasma membrane of cardiomyocytes). Statistical significance was obtained by a non-parametric one-way analysis of variance with a Kruskal–Wallis post-hoc test (*p* < 0.05). BrdU uptake (**f**, **g**, arrowheads) is significantly reduced in E11.5 mutant ventricular compact myocardium (dashed lines) (*n* = 5) as compared with stage-matched control (*n* = 5) (*p* < 0.01, 3 asterisks, **h**). The statistical significance was assessed by a t-test and a post hoc Lubischew coefficient analysis. For the quantification of BrdU-positive cells, each replicate represents the mean value from quantifications in three ventricular sections. Abbreviations: BrdU bromodeoxyuridine; CVM compact ventricular myocardium; DAPI diamidino-2-phenylindole; TnI troponin I; TVM trabeculated ventricular myocardium. Scale bars: **d**, **e**: 25 µm; **f**, **g**: 50 µm.

To identify the altered signalling pathways underlying the ventricular defects present in *G2-Itga4* mutants, we performed RNA-sequencing on microdissected ventricles from E11.5 control (*G2-Gata4*^+/+^;*G2-Itga4*^flox/+^) and mutant (*G2-Itga4*) embryos (Supplementary Fig. 2a–c). A total of 376 differentially expressed genes (DEG) (*p*.value < 1e^−3^) were identified after comparing mutant versus control samples. Out of those 376 genes, 134 were overexpressed and 242 underexpressed in the mutant tissues (Fig. 2a and Supplementary Fig. 2d, e). Gene Ontology (GO) enrichment analysis revealed functional differences between the two genotypes (Supplementary Excel File 1). Specific extracellular matrix (ECM)-related genes (*Col1a1*, *Col1a2*, *Col8a1*, *Col8a2,* or *Itga8*), which are annotated to Gene Ontology (GO) Enrichment Analysis categories such as ‘*Extracellular structure organization*’ or ‘*cell surface receptor signalling pathway*’ are significantly reduced in the mutants. These and other genes also annotate to Pathway (KEGG) Enrichment Analysis terms such as ‘*Extracellular structure organization*’ and ‘*Extracellular matrix organization*’, which were equally downregulated in mutant ventricles (Supplementary Fig. 2d, e). However, genes annotated in GO categories as ‘*Inflammatory response*’ (*Ccl6*, *Cd68*), ‘*Immune Effector Process´ (ItgaI, Vav1), ´Blood coagulation*’ (*F2rl2*, *Nbeal2*) or ‘*Wound healing*’ (*Ptpn6*, *Trim72*) were upregulated. The pathway enrichment analysis also revealed that ‘*Focal adhesion*’ and ‘*ECM-receptor interaction*’ were the most significantly enriched pathways in control animals (Fig. 2b). These data suggested that the abrogation of epicardial-myocardial cell-to-cell and cell-to-matrix interaction could, at least, partially explain the ventricular phenotype of mutants (see Fig. 1). Prompted by this finding, we investigated the status of *Vcam1* gene, encoding for the canonical myocardial receptor of α4-integrin. While no significant differences were found in the expression of *Vcam1* gene between mutant and control ventricles (Fig. 2c), the altered distribution of Vcam1 in the plasma membrane of mutant cardiomyocytes was evident (E11.5) (Fig. 2d, e). This finding indicates that anomalous Vcam1-positive cells distribution in mutant cardiomyocytes did not result from altered *Vcam1* transcription, but rather derives from a larger scale change in cardiomyocyte adhesion to the ECM or to other cells. To identify potential molecular mechanisms underlying the role of the Vcam1/α4-integrin axis in the mutant ventricular phenotype, a PPI network model analysis was conducted (see “Methods”). The analysis identified two sub-networks of protein interactions (Ezrin(Ezr)/Radixin/Moesin(Msn) and Ncf/Cybb/Rac1) that could be altered in *G2-Itga4* embryos (Supplementary Fig. 3a). Each sub-network contains proteins that potentially interact with *ITGA4 via VCAM1* (Supplementary Fig. 3b), so that the absence of epicardial ITGA4 or altered cell adhesion^35^ could affect the polarization of the cardiomyocytes.

Finally, to evaluate whether epicardial depletion in mutant hearts affected cardiomyocyte proliferation, BrdU experiments were carried out. BrdU uptake was studied in the compact ventricular myocardium of mutant embryos at E11.5 (Fig. 2f, g). This analysis revealed a significant (*p* < 0.01) decrease in cycling cardiomyocytes of the compact ventricular layer (Fig. 2h).

### Histopathology of a paediatric human CAF reveals the endocardial nature of its inner lining

**Fig. 3:**
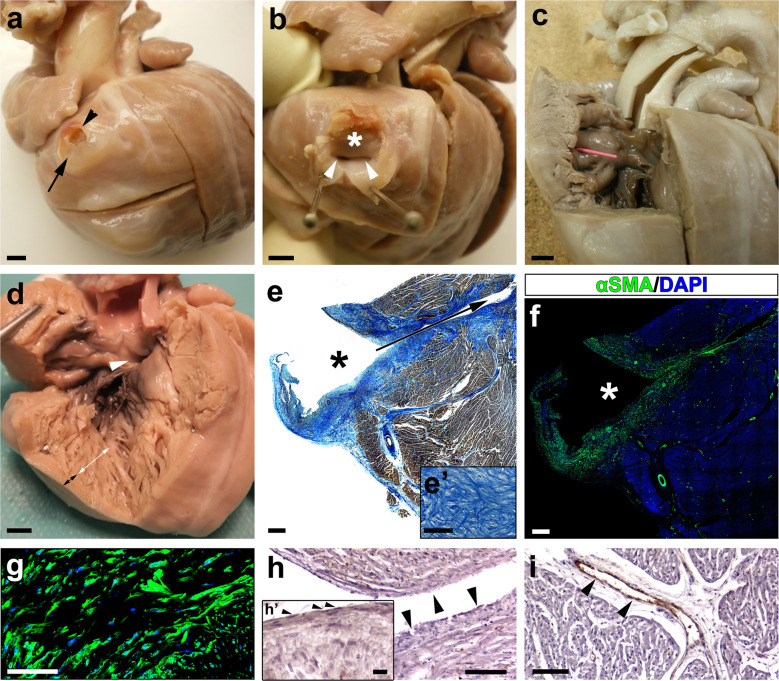
Necropsy of a paediatric CAF case supports an endocardial origin for the CAF inner lining. **a**–**c** Right lateral view of a six weeks-old human heart showing a coronary arterio-venticular communication or fistula (CAF) with a characteristic pouch-like appearance (**a**, arrow). The wall of the fistula was found to be ruptured at the time of necropsy (**a**, arrowhead). The cavity of the fistula (**b**, asterisk) shows two orifices (**b**, arrowheads) that penetrate the right ventricular wall (**b**, arrowheads) and are in continuity with the right ventricular lumen (**c**, pink probe). **d** Evidence of a large outlet ventricular septal defect (**d**, arrowhead), and a non-compacted right ventricular apex (**d**, the white double-headed arrow marks the non-compacted myocardium and the black one the compacted myocardium). **e**–**g** Mallory’s trichrome staining of histological sections from this CAF (the asterisk marks the lumen of the fistula and the arrow the communication with the ventricular lumen) shows the accumulation of ECM (**e’**) in the wall of the structure. Anti-αSMA^+^ cells are very abundant in the fistular wall (**f**, **g**). Von Willebrand factor is almost undetectable in the endothelial lining of the CAF (**h**, arrowheads; endocardial nuclei are pointed with arrowheads in **h’**), but it is conspicuous in the endothelium of the surrounding coronary vessels (**i**, brown, arrowheads). Nuclei were counterstained with DAPI (**f**, **g**; blue). Scale bars: **a**–**d**: 1 mm; **e**, **f**: 500 µm; **e’**, **g**–**i**: 100 µm; **h’**: 15 µm.

The autopsy of a six weeks-old patient who died of sudden cardiac arrest at home revealed the presence of several structural cardiac congenital anomalies, including double outlet left ventricle (DOLV) with outlet VSD, and a large coronary arterio-ventricular fistula with a pouch-like appearance (Fig. 3a, b). This CAF was found at the cardiac surface, to the right of the left anterior descending coronary artery. The outer wall of the CAF displayed a patent rupture (Fig. 3a, b), and its inner cavity was directly connected to the right ventricular lumen through two independent orifices (Fig. 3b, c). The right ventricle was poorly compacted, displaying large trabecular processes (Fig. 3d). The histological analysis of the CAF wall using Mallory’s trichrome staining showed the accumulation of ECM proteins in the wall of the structure (Fig. 3e). ECM accumulation overlapped with the presence of large, compacted numbers of smooth muscle cells in the CAF wall and the medial layer of associated coronary arteries (Fig. 3f, g). To determine whether the CAF was lined internally by endothelial cells the expression of Von Willebrand (VW) factor was considered. Anti-VW immunohistochemistry indicated little or no immunoreactivity for this marker in the tissue covering the CAF lumen (Fig. 3h), whereas significant amounts of VW protein were detected in the endothelium of adjacent coronary vessels (Fig. 3i).

### Cryoinjury of the avian embryonic heart disrupts ventricular myocardial continuity and results in CAF-like structures

**Fig. 4:**
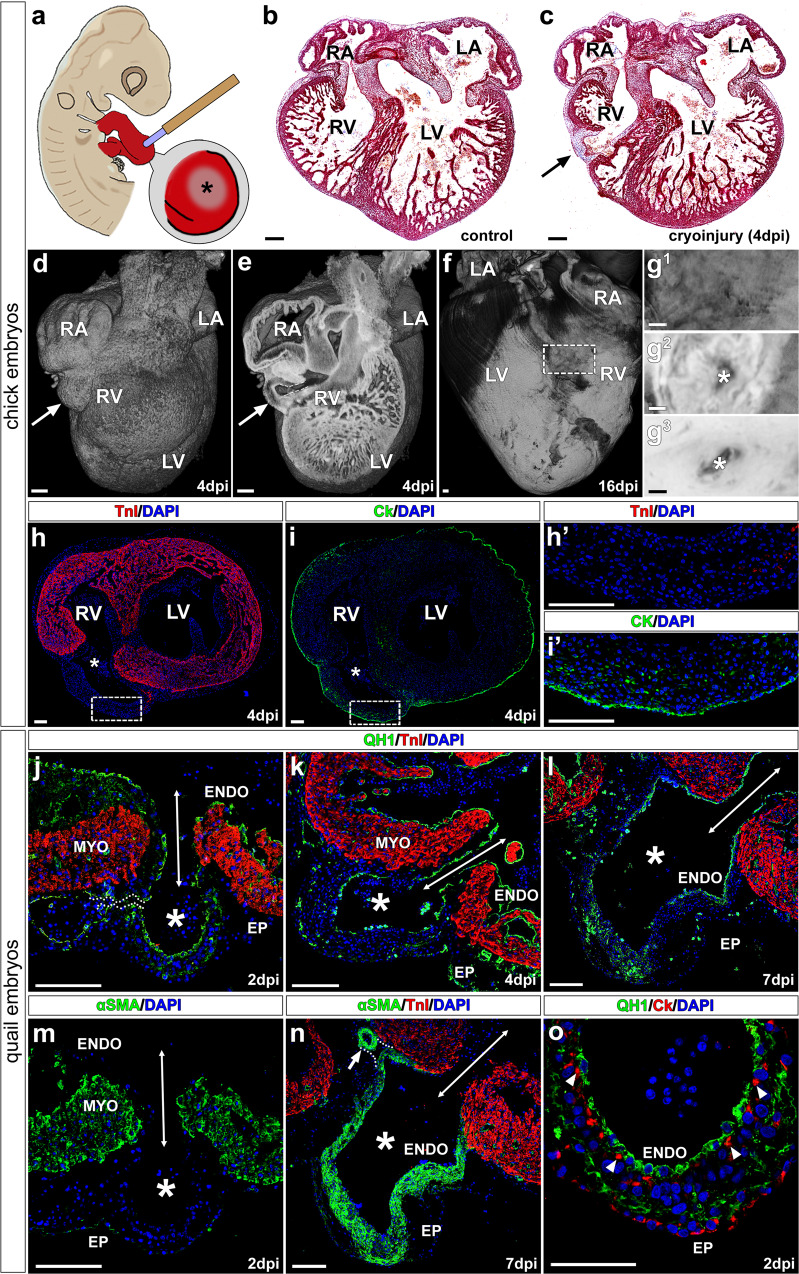
Local cardiomyocyte damage disrupts ventricular myocardial continuity and results in endocardial extrusion towards the pericardial cavity. **a** Chick embryos were submitted to ventricular cryoinjury at HH16-17 stages. Frontal (cranial) sections of control (**b**) and cryodamaged (**c**) chick. The damaged area in the cryoinjured ventricular wall (4 days post injury) is marked with an arrow. **d**–**g**^**3**^ HREM reconstructions of cryoinjured chick embryonic hearts. At 4 days post injury (dpi), a pouch-like structure is formed in the damaged area (**d**, **e**, arrows) directly connected to the ventricle lumen (**e**, dashed line). These kind of structures remain visible at 16 dpi (**f**, **g**). Transmural analysis of the ventricle shows that the lumen of this fistular vesicle is in communication with the ventricular one (**g**^**1**^−**g**^**3**^, asterisks). **h**, **i** Myocardial ventricular discontinuities (asterisks) are TnI^−^ (**h**, **h’**) but are externally lined by Ck^+^ tissue (**i**, **i’**). **j**–**o** Cryocauterisation of quail hearts results in the formation of pouch-like structures forming at the damage site (**j**–**o**, asterisks); their lumen is continuous with the ventricular one (**j**–**n**, double headed arrows), and in some cases, can be continuous with the endothelium of normal coronary vessels (**j**, **n**, dotted lines). The quail specific marker QH1 labels the embryonic endocardium and vascular endothelium (**j**–**l**, **o**). At 2dpi, alpha smooth muscle actin (αSMA) expressed in the immature ventricular myocardium (**m**). αSMA is mainly observed in the wall of fistula-like structures at 7 dpi (**n**) and the tunica media of adjacent coronary arteries (**n**, arrow). Endocardial (QH1^+^)-epicardial (Ck^+^) contact takes place early in the damaged area (2 dpi, O, arrowheads). Abbreviations: αSMA alpha smooth muscle actin; CK cytokeratin; DAPI diamidino-2-phenylindole; dpi days post injury; ENDO endocardium; EP epicardium; LA Left atrium; LV left ventricle; MYO myocardium; RA Right atrium; RV right ventricle; TNI troponin I. Scale bars: **b**–**g**: 200 μm; **h**, **h’**, **i**, **i’**, **j**–**o**: 100 μm.

Since *G2-Itga4* mouse mutant embryos died shortly after endocardial vesicles form on the heart surface (see Fig. 1n), a cardiac cryoinjury method was applied to chick embryonic hearts to better understand the cellular mechanisms underlying the formation of the fistula wall (Fig. 4a). The local cryoinjury of the embryonic ventricular myocardium resulted in the appearance of a single myocardial discontinuity and the formation of CAF-like structures on the ventricular surface (Fig. 4b, c and Supplementary Fig. 4a–c). A 3D analysis of chick cryodamaged hearts at 4 and 16 days post-injury (dpi) (corresponding to HH46 stage, around 1 day before hatching), revealed the prevalence of a thin channel (fistula) connecting the pouch-like structure with the ventricular lumen (Fig. 4d–g). Contact between the pouch-like vascular lining and the endothelium of neighbouring coronary vessels may also occur (Fig. 4j, n), although this is a far less common feature in these avian CAF. Immunohistochemical analysis of these structures showed they are limited by an external wall including cytokeratin^+^ epicardial epithelial cells and epicardial-derived mesenchyme (Fig. 4h–i’).

In order to characterize the CAF-like endothelial lining, the heart cryoinjury method was also applied to quail embryos, and the QH1 quail endothelial marker was used to counterstain endothelial cells (including endocardial ones) in the developing heart (Fig. 4j–l). Three developmental stages of quail embryos were analyzed, namely 2-, 4- and 7-dpi (Fig. 4j–o). As described for chick embryos (a species for which no bona fide endothelial markers are available), the continuity of the myocardium was locally lost in cryoinjured quail embryos as illustrated by the absence of troponin (TNI) (Fig. 4j) and alpha smooth muscle actin (αSMA) staining in the damaged area (Fig. 4m). A local extrusion of putative endocardial and epicardial tissues towards the pericardial cavity was observed in all cryodamaged hearts (Fig. 4j–o). Although at 2 dpi αSMA expression can still be found in the embryonic myocardium (Fig. 4m), αSMA cardiac becomes progressively restricted to the smooth muscle cells of the developing coronary arteries and the forming wall of CAF-like structures at 4 and 7 dpi (Fig. 4n, o). At 2dpi, the extruded endocardium was in contact with some epicardial cells (CK^+^) (Fig. 4o).

### Experimental avian fistulae originate from epicardial-derived and endocardial cells

**Fig. 5:**
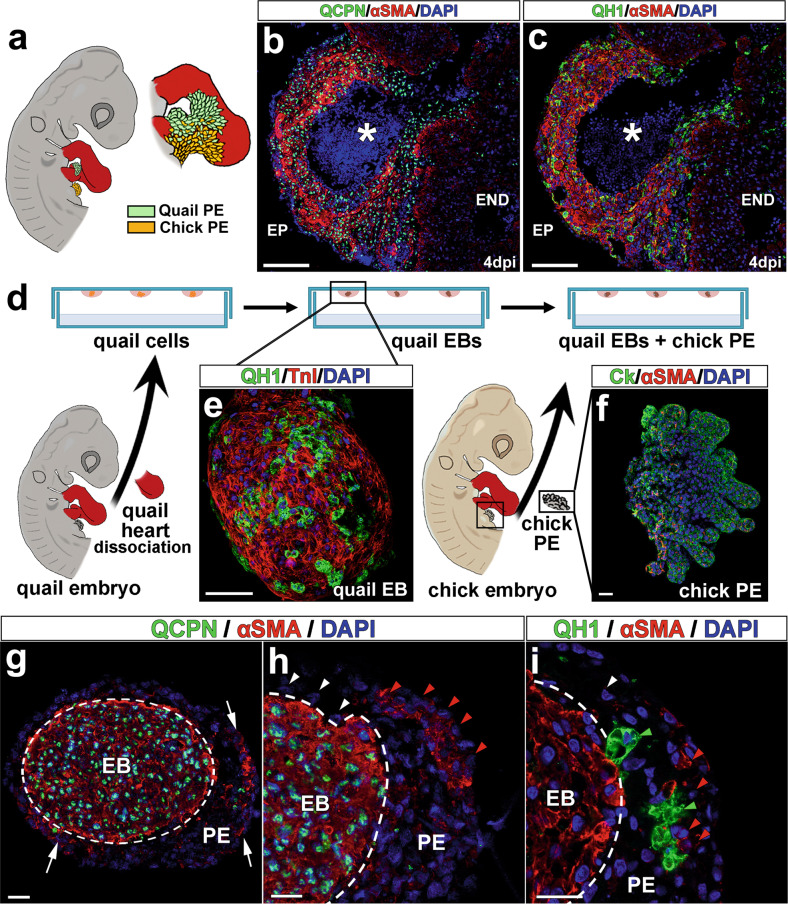
Cellular origins in avian CAF-like structures. **a** Quail-to-chick proepicardial chimeras were first constructed by grafting a quail proepicardium close to a chick cryodamaged heart and then submitted to cryocauterisation as shown (Fig. 4a). All donor (quail) proepicardial-derived cells are QCPN^+^ (**b**, green fluorescent nuclei) and quail-derived endocardial/endothelial cells are QH1^+^ (**c**, green fluorescent cells). **d**–**g** Co-culture of quail endocardial and chick epicardial tissues. Quail heart dissociation and subsequent hanging drop culture allows for the externalization of quail endothelial cells on the surface of forming embryonic bodies (**e**, QH1^+^, green fluorescent cells). Co-culture of these embryonic bodies with freshly excised epicardial progenitor (proepicardial) cells (**f**, Ck^+^/αSMA^−^, green fluorescent cells) results in the rapid differentiation of αSMA^+^ cells from this latter tissue (**g**, arrows). Chick proepicardial cells (QCPN^−^, blue fluorescent nuclei) cover quail-derived tissues after 1 day of culture (**h**, dashed line). These proepicardial-derived cells mostly are αSMA^−^ (**h’**, white arrowheads), but some of them are αSMA^+^ (**h**, red arrowheads). These αSMA^+^ cells (**i**, red arrowheads) are adjacent to quail endothelial (QH1^+^) cells (**i**, green arrowheads). Quail immature myocardium is always αSMA^+^/QCPN^+^ (**g**, **h**, green fluorescent cell nuclei, red fluorescent cytoplasm). Abbreviations: αSMA, alpha smooth muscle actin; Ck cytokeratin; DAPI diamidino-2-phenylindole; EB embryonic body; PE proepicardium. Scale bars: **b**, **c**: 100 μm; **e**–**i**: 25 μm.

Interspecific quail-to-chick transplantations have for long BEEN regarded as a reliable method to permanently trace the fate of embryonic tissues^19^ (Fig. 5a). Hence, we used this approach to evaluate whether CAF share, at least in part, an epicardial origin with coronary blood vessels^18^. Proepicardial (PE) quail transplantations into chick hosts and subsequent cryocauterisation of the developing chimeric embryo revealed that the majority of smooth muscle cells (αSMA^+^) forming the CAF-like wall were donor-derived (quail) cells expressing the quail-specific QCPN nuclear marker (Fig. 5b). The absence of this QCPN marker (Fig. 5b) or the quail-specific endothelial marker QH1 (Fig. 5c) in the inner lining of CAF-like structures (which is continuous with the host chick endocardium), suggests an endocardial origin for these cells and rules out PE endothelial contribution to these anomalies.

Finally, to test whether early epicardial cell contact with the embryonic endocardium accelerates smooth muscle differentiation, thus contributing to CAF-like wall formation, interspecific co-culture analyses of chick epicardial progenitor (proepicardium) and quail myocardial-endocardial tissues were developed. Hanging drop culture of quail embryonic hearts (Fig. 5d) allowed for endocardial (QH1^+^) growth and accumulation at the surface of the cardiac explant (Fig. 5e). Chimeric constructs were formed by aggregating chick proepicardia (Fig. 5f) to these quail explants for 24 h (Fig. 5g–i). After immunohistochemical inspection, the alpha-smooth muscle actin (αSMA) marker was observed in chick (QCPN^−^) proepicardial-derived cells in close association with quail endocardial (QH1^+^) cells. αSMA marker expression was scarce in areas where chick cells were in contact with myocardial (αSMA^+^/QH1^−^) but not endocardial (αSMA^-^/QH1^+^) cells (Fig. 5g–i).

## Discussion

Aberrant connections between coronary arteries and a heart chamber or a large vessel, commonly referred-to as coronary arterio-ventricular fistulae (CAFs)^6^, have been known for more than a century. These anomalies are rare, but often associate to multiple cardiac complications such as myocardial hypertrophy, endocarditis, heart dilatation, and cardiac failure^4,7,8^.

A certain degree of confusion exists in the literature about the origin of CAFs. This is, at least in part, due to semantics, since the terms coronary ‘aneurysm’ and ‘pseudoaneurysm’ are often interchangeably used to refer to CAF^5,36^. This requires a primary distinction between anomalies of the coronary wall with an acquired origin, which may result from defective surgical procedures^37^, and congenital CAF anomalies with a developmental origin^7^. A note on the nature of CAF should also be made as based on strict histopathological criteria, as true congenital vascular aneurysms involve the local bulging of the three tissue layers of a vessel (intima, media, and adventitia), whereas pseudoaneurysms (or false aneurysms) are anomalous structures with a characteristic pouch-like appearance containing tissue from the medial and adventitial mural layers only^38^. In our work, we have revisited the histoarchitecture of human CAF through the histological analysis of a conspicuous CAF found in a six-week-old child. This examination indicates that endothelial (intimal), smooth muscle (medial), and fibroblastic (adventitial) tissues form the wall of these structures. Detailed reports indicate that the wall in human CAFs contains smooth (but not striated) muscle bundles frequently separated by several internal elastic laminae, as well as a characteristic intimal layer showing non-specific fibrous thickening^39^. Similar histological features were found in the paediatric case reported in our study: the internal surface of the CAF is lined by a vascular endothelium displaying a characteristically poor Von Willebrand immunoreactivity associated to the endocardium^40^, with the majority of the fistula wall formed by smooth muscle cells (α-SMA^+^). On a final note, our analysis of this human case revealed that the ventricular myocardium was poorly compacted. This finding developmentally associates poor ventricular thickening or compaction^41^ with the occurrence of congenital CAF as described in paediatric and adult human patients^42^.

While the congenital origin of CAFs is widely accepted^43^, no data on the disrupted developmental mechanisms that lead to CAF formation are available in the literature. Although the anatomical description of CAF may suggest these anomalies are secondary to disrupted coronary vasculature morphogenesis^44^, it has also been suggested that they could form by the persistence of sinusoidal connections between the forming coronary arteries and the primitive tubular heart lumen^45^. These two hypotheses, reinterpreted in light of recent discoveries on the embryonic origin of coronary blood vessels^16–18^, are fully compatible and could explain the origin of part of congenital CAF. Indeed, connections between both the embryonic *sinus venosus* at the venous pole of the heart and the ventricular lumen with the primitive coronary capillary plexus occurs during normal coronary embryonic development^16–18^. Such connections are progressively lost as the ventricles thicken and mature during the perinatal stages^46^. Thus, the proper growth of ventricular myocardium is necessary to maintain the patterning and stabilization of embryonic coronary vessels and is also required to separate coronary endothelium from its endocardial origin. In this work, we suggest that the anomalous disruption of compact myocardial wall formation may result in persistent communications between coronary blood vessels and the cardiac lumen leading to CAF formation.

To test our working hypothesis, we used two animal models. The first one is a mouse with an epicardial deletion of the *Itga4* gene. This conditional mutant recapitulates the severe ventricular compact myocardial layer defects, known to be secondary to the anomalous development of the epicardium, found in *Itga4* systemic mutants^27^, but survive beyond embryonic day 9.5. Relevant to our approach, *Itga4* mutants represent an extreme case of cell (Itga4) and non-cell autonomous (Vcam1) defects associated to defective epicardial development, a key developmental process required for both coronary blood vessels and ventricular wall formation^23^.

Our second animal model is the chicken embryo, which is amenable to a large variety of *in ovo* experimental manipulations with little or no impact on embryo viability^47^. In particular, we have taken advantage of a recently described procedure allowing for the creation of restricted damage on embryonic tissues by means of local cryocauterisation^34^. In this case, cryocauterisation was used to locally disrupt ventricular myocardial wall continuity prior to the formation of the epicardium. In this experimental setting, the epicardium remains unaffected, allowing for the study of epicardial mesenchymal derivatives in the damage area where CAF will form.

Genetic deletion of the *Itga4* gene in mouse epicardial progenitors and epicardial cells disrupts early epicardial development, significantly reducing the number of epicardial cells as shown by histological and epicardial-specific gene expression analyses. Abnormal epicardial development in these mutants is concomitant with the lack of thickening of the ventricular compact myocardial layer due to reduced cardiomyocyte proliferation, as it has been described for other epicardial mutants^48^. Such discontinuities allow for the extrusion of the endocardium, which can be distinguished from the forming coronary vascular endothelium by its strong endomucin expression. Indeed, endomucin marker expression confirms the endocardial origin of the tissue lining the CAF cavity, also suggesting it does not derive from the differentiating coronary vascular endothelium found in the cardiac surface (endomucin-negative).

The transcriptomic analysis of epicardial *Itga4* mutants revealed a significant increase in genes associated to tissue damage (thrombosis, blood clot formation, inflammation pathways) as well as a marked decrease in genes linked to cell surface signalling and ECM organization. These findings, taken together, strongly suggest that epicardial-to-myocardial adhesion mechanisms, including those mediated by the ECM, were affected by *Itga4* deletion. Consequently, we checked the status of *Vcam*-1, the canonical α-4 integrin epicardial ligand^49^. Interestingly, *Vcam-1* gene expression level was unchanged in *Itga4* epicardial mutants, but Vcam1 protein distribution in the plasma membrane of cardiomyocytes was found to be severely altered in these animals. Both the reduced number of epicardial differentiating cells and the altered secretion of subepicardial matrix proteins in mutant embryos could explain the poor cell surface organization of Vcam1. Our in silico protein interaction modelling suggested that different proteins belonging both the Ezrin/Radixin/Moesin and Ncf/Cybb/Rac1 pathways could potentially interact with Vcam1. It is tempting to think these pathways could be involved in the developmental origin of the mutant phenotype, so that disruption of ventricular cardiomyocyte polarity may have an impact on ventricular cardiomyocyte cohesiveness and this, in turn, result in myocardial discontinuities. This hypothesis, however, needs a further, detailed analysis, as chamber cardiomyocyte polarization is far from been complete at the earliest embryonic stages studied in our work (E9.5). The specific cellular context of genes related to *Itga4* is suggested by the high prevalence of epithelial (and epicardial) specific proteins in the model. Moreover, our model also suggests the existence of a biochemical/functional relationship between Itga3 and Itga4. Whether anomalies in Itga4-Itga3 protein interactions could promote the previously described CAF-like phenotype needs further experimental analysis. Taken together, these results indicate that defective cell-to-cell and cell-to-matrix adhesion could underlie the appearance of the characteristic ventricular myocardial discontinuities of epicardial *Itga4* mutants. In turn, these ventricular myocardial wall discontinuities could represent a passage through the compact myocardium for the endocardium, providing a physical continuum between the endocardial lining of the cardiac chamber with the pericardial space.

The death of *Itga4* epicardial mouse mutants at E12.5–13 prevented us from studying the formation of the CAF-like structures in these animals in more detail. To progress in our analysis of these anomalies, we decided to use chick embryos, a highly manipulable experimental animal model extensively used to study embryogenesis. Hence, we carried out limited cryocauterisation of the chick embryonic heart (HH16–17, around 60 h of incubation) to reproduce the ventricular myocardial discontinuities we identified in *Itga4* epicardial mutants. Our results show that the mechanical disruption of the chick embryonic myocardium faithfully reproduces the formation of arterio-ventricular anomalies showing a structure very similar to that described for human CAF. The combination of cryocauterisation and interspecific quail proepicardium-to-chick chimerisation for cell fate tracking demonstrates that the smooth muscle wall covering these anomalies, as that of coronary arteries, has epicardial origin^23^. Our results also confirm that the inner lining of the fistula cavity is not an epicardial derivative; the absence of the QCPN quail donor marker and the continuity of these cells with the ventricular endocardium strongly suggest a host endocardial origin for this tissue. The vascular lining of the fistula, which is always continuous with the endocardial ventricular lumen, can appear connected with the endothelium of close coronary vessels, although this feature is not frequent in our samples. This may be explained by the early formation of the pouch-like structure, that seems to derive from the ventricular endocardium rather than from the coronary endothelium. Therefore, formation and muscularisation of the CAF seems to precede coronary blood vessel formation, precluding early connection between the pouch-like lumen and coronary vascular endothelium. Since coronary smooth muscle is known to be a major embryonic epicardium derivative^23^, and endothelial-to-mesodermal progenitor cell contact and paracrine molecular cross-talk efficiently result in smooth muscle differentiation^50^, we tested in vitro whether early endocardium-to-epicardial-derived cell contact could effectively induce smooth muscle differentiation from the latter cell type. Our results convincingly demonstrate that early in vitro endocardium-to-epicardial-derived mesenchymal cell interaction triggers epicardial cells differentiation into smooth muscle cells, as normally happens in vivo.

**Fig. 6:**
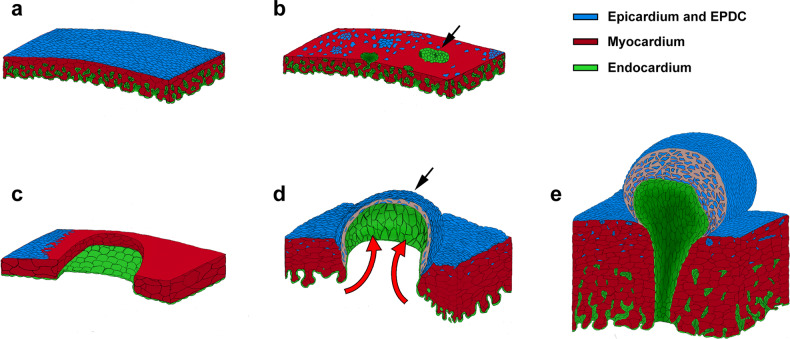
A developmental model for cells dynamics during CAF formation. Formation of the mouse epicardium in *Itga4* mutants (**b**) is disrupted as compared with the control condition (**a**). Loss of epicardial tissue reduces myocardial proliferation and results in ventricular myocardial wall discontinuities, local endocardial extrusion (**b**, arrow) and leads to embryonic death. **c**–**e** In avian embryos, cryocauterisation-induced myocardial discontinuities precede epicardial formation and growth, which is originally normal. Blood pressure (**d**, red arrows) pushes and extrudes the endocardium towards the pericardial cavity, forming a pouch-like structure that resembles a single CAF (**d**, black arrow). Precocious endocardial-epicardial contact promotes the differentiation of smooth muscle and fibroblastic cells from epicardial tissues (**e**, blue), which were originally fated to contribute to the coronary vascular system. Green, endocardium; blue, epicardium; red, myocardium.

We have herein shown that myocardial discontinuities in the embryonic ventricular wall, either secondary to anomalous epicardial development or resulting from a direct damage to the embryonic myocardium, cause CAF in two different vertebrate models via the extrusion of the endocardial lining of the developing heart, the precocious and anomalous formation of a smooth muscle wall over the extruded endocardium, and the persistence of arterial to ventricular lumen connection (Fig. 6). The histological analysis of a human CAF, compared to our cell-tracing experiments, confirm the nature and origin of the tissues that form this congenital CA anomaly. The experimental analysis of coronary and ventricular wall morphogenesis in two classical animal models for the study of cardiovascular development (mouse and chick) reveals significant similarities in the phenotypes resulting from the combined disruption of epicardial and myocardial normal development that, in turn, strikingly reproduce human coronary arterio-ventricular fistulae. To the best of our knowledge, these results are one of the very few examples on the origin of a specific CHD to be found in the literature, providing clues to the identification of potential genetic markers for the diagnosis of such conditions.

## Supplementary information

Supplementary material
